# Glymphatic-System Function Is Associated with Addiction and Relapse in Heroin Dependents Undergoing Methadone Maintenance Treatment

**DOI:** 10.3390/brainsci13091292

**Published:** 2023-09-07

**Authors:** Lei Wang, Yue Qin, Xiaoshi Li, Xin Li, Yuwei Liu, Wei Li, Yarong Wang

**Affiliations:** 1 Department of Radiology, The First Affiliated Hospital of Xi’an Jiaotong University, Xi’an 710061, China; shagojo@163.com (L.W.); qinyuemr@126.com (Y.Q.); 2 Department of Radiology, Xi’an Daxing Hospital, Xi’an 710016, China; 3 Department of Medical Imaging, People’s Hospital of Tongchuan City, Tongchuan 727000, China; 4 Department of Radiology, Tangdu Hospital, Air Force Military Medical University, Xi’an 710038, China

**Keywords:** DTI-ALPS, methadone maintenance treatment (MMT), heroin dependence, glymphatic system

## Abstract

This study investigates the impact of methadone maintenance treatment (MMT) on the brain glymphatic system (GS) in opioid addiction in China. A total of 51 male MMT patients, 48 demographically matched healthy controls (HCs), and 20 heroin dependents (HDs) were recruited for this study. The GS functioning was assessed using diffusion-tensor-imaging analysis along perivascular spaces (DTI-ALPS index) and the bilateral ALPS divergency (DivALPS). Group differences were analyzed utilizing ANOVA and two-sample *t*-tests. The relationship between DivALPS and relapse rate was explored using regression analysis. The DTI-ALPS index was significantly higher for the left-side brain than the right side in all three groups. There was a significant difference for the right side (*p* = 0.0098) between the groups. The MMT and HD groups showed significantly higher DTI-ALPS than the HC group (*p* = 0.018 and 0.016, respectively). The DivALPS varied significantly among the three groups (*p* = 0.04), with the HD group showing the lowest and the HC group the highest values. Significant negative relationships were found between relapse count, DivALPS (*p* < 0.0001, Exp(B) = 0.6047), and age (*p* < 0.0001, Exp(B) = 0.9142). The findings suggest that MMT may contribute to promoting brain GS recovery in heroin addicts, and modulation of the GS may serve as a potential biomarker for relapse risk, providing insights into novel therapeutic strategies.

## 1. Introduction

Heroin dependence is a challenging issue with profound implications for individuals and society. Methadone maintenance treatment (MMT) has proven to be an effective intervention in managing heroin dependence, as it reduces opioid use and supports long-term recovery. The heroin-to-methadone swap program, also known as opioid substitution treatment, offers several benefits. Methadone is a long-acting opioid agonist that helps reduce withdrawal symptoms and cravings associated with heroin use. This approach provides a controlled and supervised environment for individuals to gradually transition away from heroin, reducing the risks of illicit drug use, overdose, and transmission of infectious diseases [1]. However, there are risks associated with methadone treatment as well. Methadone can cause its own dependence, and improper dosing or administration can lead to overdose [1,2]. Additionally, some individuals may misuse or divert methadone. Psychological and social factors may also influence the success of the treatment [3]. The Chinese MMT program has been effective in reducing the number of injections, injection-related risk behaviors, and adversities due to HIV/HCV infection and drug-related harm among drug users [4]. However, several factors—such as low methadone dosage and concurrent use of heroin [5]—have been identified in the literature as associated with MMT dropout [6,7]. One-year MMT retention rates in China ranged from 30% to 86%, and most dropouts occurred within the first 12 months of the treatment [8]. Therefore, there is an urgent need to understand the underlying neural mechanisms of an MMT participant’s relapse behavior. It is essential to understand the effects of MMT on the brain of heroin-dependent (HD) individuals. 

Neuroimaging techniques have been used to search for neuronal substrates associated with MMT outcomes. Functional and structural alterations due to drug abuse and the associations between relapse behavior and neural alterations have been reported by many studies. Using functional connectivity-based methods, studies suggested the disrupted coupling between the salience network and default mode network (DMN) was associated with relapse behavior [9] and the potential predictive value of the DMN concerning heroin relapse under MMT [10]. Furthermore, white-matter microstructure indices in the posterior limb of the internal capsule were addressed for their association with relapse to heroin use in MMT [11]. Recently, a data-driven method was used to identify the poor and good MMT responder’s prognosis [12] based on the network graph measures. The above studies mainly focused on brain parenchyma; however, little research has been done on the role of the brain interstitium in addiction and relapse, and the mechanisms involved are not well understood.

The glymphatic system (GS), discovered by Iliff et al. [13,14], comprises cerebrovascular aquaporin-4 (AQP4) located at the end of astrocytes and in the cerebral perivascular space (PVS). Similar to the lymphatic system, the GS clears metabolic waste and is considered a refined clearance mechanism of the central nervous system [15]. Convective interstitial-fluid (ISF) bulk flow propels waste products towards perivenous space, where they are drained from the brain through the cervical lymphatic system. The GS removes various metabolites, including soluble amyloid beta (Aβ), proteins, lipids, proinflammatory cytokines, and neurotoxic solutes [13,16,17]. Animal and human studies have shown that alcoholism [18] and cocaine use [19] impair glymphatic function and reduce the clearance of brain metabolites. The effect of opioid drugs on human brain GS function remains unclear due to its complex effects. On the one hand, drug abuse may disrupt the blood–brain barrier (BBB) [20]. On the other hand, drug abuse could induce neuroinflammation [21] and damage the microtubules of the brain GS but also activate the brain GS to enhance the clearance of brain metabolic waste and neuron debris, thereby improving the function of brain GS. The main clearance pathway of methadone in the human brain is through the GS [22]. Moreover, the effects of methadone and heroin are dependent on various factors, including dosage, drug concentration, and the duration of receptor site occupancy. Disruptions in GS function have the potential to influence the pharmacokinetics of these drugs, affecting their duration of action and overall effectiveness. Despite the complexity of these mechanisms, the connection between methadone’s impact on the GS, and its clearance process, and the overall therapeutic effectiveness on relapse risk remains unclear.

There are various MRI approaches available to investigate the GS function, including dynamic contrast-enhanced MRI (DCE-MRI) with gadolinium-based contrast [23], DWI-based methods utilizing the apparent diffusion coefficient (ADC) [24], and diffusion kurtosis measures [25]. However, it is important to note that these methods may require invasive procedures or have specific scanning protocols and processing steps. Taoka and colleagues have recently proposed a new technique for assessing the function of the human GS using diffusion-tensor-imaging analysis along the perivascular space (DTI-ALPS index) [26,27]. This novel method harnesses the spatial relationship between fiber tracts and the perivascular space at the corona radiata and determines the ratio of diffusivity in the direction of the perivascular space versus that perpendicular to the primary fiber tract and perivascular space. The DTI-ALPS index holds promise in several fields of neuroscience research, including the evaluation of neurodegenerative diseases and other neurological disorders, such as Parkinson’s disease [28], stroke [29], Alzheimer’s disease [30], and type 2 diabetes mellitus [31]. Being an in vivo method, it is also suitable for investigating the brain GS of patients in the MMT program.

The aim of this study is to examine the relationship between drug use and alterations in GS functionality in individuals undergoing MMT. We intend to utilize DTI-ALPS analysis to (1) investigate the impact of prolonged opioid use on GS function and evaluate the potential rehabilitative effects of MMT on these alterations and (2) explore the association between relapse behavior and the GS functioning to assess whether the clearance process of methadone influences treatment outcomes.

## 2. Materials and Methods

### 2.1. Participants

Fifty-one male heroin-dependent individuals undergoing MMT, with a mean age of 35.76 years (age range: 22–53) and MMT duration of 5–56 months, were recruited from the Outpatient of Xi’an methadone substitution treatment center for the MMT group. The daily effective dosage of methadone ranged from 20 to 79 mg. The MMT program follows the “Guidelines for Diagnosis and Treatment of Opioid Use Disorder” in China (http://www.nhc.gov.cn/yzygj/s3573/201712/d97b1676239542eea151227bc993315b.shtml, accessed date: 9 June 2023). Forty-eight male healthy controls (HC) with a mean age of 35.42 years (age range: 19–48) were recruited from the local community through advertisements. The HC group was well matched with the MMT group in terms of age and education level and had no history of substance abuse except for nicotine. Additionally, we recruited 20 heroin-dependent individuals (HD) with a mean age of 31.5 years (age range: 22–47) who had undergone detoxification and had not received methadone treatment. To match the HD group in terms of age and education level, 33 MMT participants with a mean age of 30.91 years (age range: 22–40) and 33 HC participants with a mean age of 31.39 years (age range: 19–41) were selected from the MMT and HC groups, respectively. Detailed information can be found in Table 1.

The inclusive criteria for participation in the HD and MMT groups were (1) meeting DSM-IV-TR criteria for heroin dependence and (2) being right-handed according to the Edinburgh Handedness Inventory. For the MMT group, an additional inclusive criterion was that the participants should have been receiving MMT for at least three months and a stable dose for at least one month before baseline. Exclusion criteria for all participants included DSM-IV-TR Axis I disorders, brain trauma, history of head trauma or neurological disease, current medical illness, and any contraindication during MRI examination. Further details on the participants can be found in Table 1. In the present study, only a small number of participants were left-handed, while the majority were right-handed. To control for the confounding factor of handedness, we decided to include only the right-handed participants.

This study was approved by the Ethics Committee of the Fourth Military Medical University’s Tangdu Hospital (No: TDLL-2014087), and all participants provided written informed consent.

### 2.2. MRI Acquisition

At the time of recruitment, all participants received magnetic resonance imaging (MRI) using the GE Signa Excite HD 3.0 Tesla MR scanner at Tangdu Hospital. Routine T2-weighted images were acquired to exclude any gross structural abnormalities. We used a spin-echo echo-planar sequence for diffusion-tensor-imaging (DTI) data acquisition, with a repetition time of 7600 ms, echo time of 61.5 ms, 25 gradient orientations, matrix size of 128 × 128, field of view of 240 × 240 mm, 2 excitations, 4 mm slice thickness with no gap, and b-value of 1000 s/mm^2^. Each participant’s total scanning time was approximately 10 min. Foam padding was used to minimize head movement, and earplugs were provided to reduce the noise during scanning and enhance the comfort of the participant during the scanning procedure.

### 2.3. Follow-Up Relapse Evaluation

Previous studies have demonstrated that most cases of dropout and relapse in opioid-dependent MMT patients occur within 12–60 months after MMT initiation [6,32]. Therefore, we set the follow-up duration for this study to 24 months. Following the MRI scan, MMT participants underwent monthly structured interviews and urine drug tests to assess substance use during the follow-up period. In China, opiates (primarily heroin, 37.4%) and synthetic drugs (mainly methamphetamine, 53.4%) are the most commonly abused drugs (Report on China’s Drug Situation in 2021, http://newyork.china-consulate.gov.cn/eng/xw/202209/t20220901_10759286.htm, accessed date: 12 June 2023). We defined relapse as any use of heroin or methamphetamine identified through a positive urine drug test (Morphine/Methamphetamine Diagnostic Kit, Guangzhou Jianlun Biological Technology Co., Ltd., Guangzhou, China). Participants who missed their appointments without contacting the study coordinator were also considered to have relapsed.

### 2.4. Imaging Data Processing

DTI data were processed using the FSL package [33] (version 6.0.6.2, https://fsl.fmrib.ox.ac.uk/fsl/fslwiki). Eddy current and motion correction were done using the eddy correction tool in FSL. The brain extraction tool (BET) was used to remove scalp and skull from the head and generate brain masks. Water diffusivity along the x, y, and z axes (the Dxx, Dyy, and Dzz maps) as well as the factional anisotropy (FA) maps were estimated from the eddy-corrected data and rotated b-vectors using DTIFIT. After tensor fitting, the tract-based skeleton statistic (TBSS) routine was used to register FA maps from each participant into a human-connectome project FA atlas, which is in Montreal Neurological Institute (MNI) space. Then, all the diffusivity maps were registered into the same space using TBSS non-FA scripts, which extracts the affine matrices and warp fields estimated from FA registering and applies them to these diffusivity maps. 

**Figure 1:**
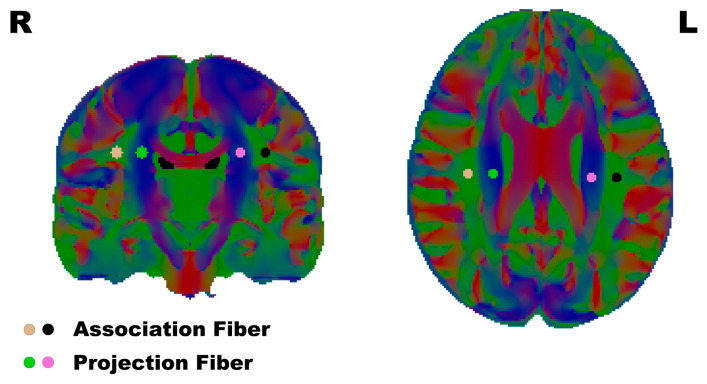
The ROI were placed in the association fibers and projection fibers according to the reference papers. L/R: Left/Right.

To calculate the DTI-ALPS, regions of interest (ROI) were defined as 3 mm radius spheres, and the positions of the ROI were placed in the projection- and association-fiber regions of the bilateral hemisphere on the color-FA map in the horizontal plane of the lateral ventricle body, the same as previous DTI-ALPS research [29] (Figure 1). 

The DTI-ALPS index was calculated as follows:

(1) $$\mathrm{DTI}-\mathrm{ALPS}=\frac{mean\;\left(D_{xx-proj}\;,\;D_{xx-assoc}\right)}{mean\;\left(D_{yy-proj},\;D_{zz-assoc}\right)}$$

Here, $D_{xx-proj}$, $D_{xx-assoc}$, $D_{yy-proj}$ and $D_{zz-assoc}$ are the mean diffusivity in the ROI placed in projection fibers and the association fibers along the x-axis, y-axis, and z-axis, respectively. 

In addition to the calculation of the traditional bilateral DTI-ALPS index, we defined a new index for evaluating the functional differences between the individual’s left and right brain GS—the normalized bilateral DTI-ALPS divergence index (DivALPS). This DivALPS index was calculated using the following formula:

(2) $$DivALPS=\frac{{ALPS}_{L}-{ALPS}_{R}}{{ALPS}_{L}+{ALPS}_{R}}$$

This formula was defined similarly by Zhang et al. [34]. 

### 2.5. Statistics

R version 4.2.3 (https://cran.r-project.org/) was used to perform demographic data analysis, intergroup comparison, and regression analysis.

The differences between the MMT and HC groups and between MMT and HD groups were analyzed using the two-sample *t*-test for continuous data. The differences among the 3 groups were analyzed using one-way ANOVA. 

To reveal the relationship between brain glymphatic function and relapse behavior of the MMT participants, Poisson regression with the robust method (R “robust” package) was utilized. The robust Poisson regression method has been enhanced to address potential outliers and influential points while diminishing reliance on the assumption of a Poisson distribution, thereby strengthening the rigor of the analytic results [35,36,37]. In the regression model, the normalized divergence of bilateral DTI-ALPS was defined as the variable of interest, age as the covariates that influence both the relapse behavior [12] and brain glymphatic function [38,39], and relapse count from the 24-month follow-up as the dependent variable.

## 3. Results

### 3.1. Demographic and Clinical Characteristics

There were no significant differences in age, education, and smoking between 51 MMT and 48 HC participants, as well as among the 33 MMT, 33 HC, and 20 HD participants (*p* > 0.05). The BDI score of the MMT group was higher than that of the HC group, while it showed no significant difference compared with that of the HD group. The craving score (calculated as the craving score after cue-picture showing minus baseline craving score) was significantly higher in the HD group than MMT group. Detailed information can be found in Table 1.

### 3.2. The DTI-ALPS of Three Groups

**Figure 2:**
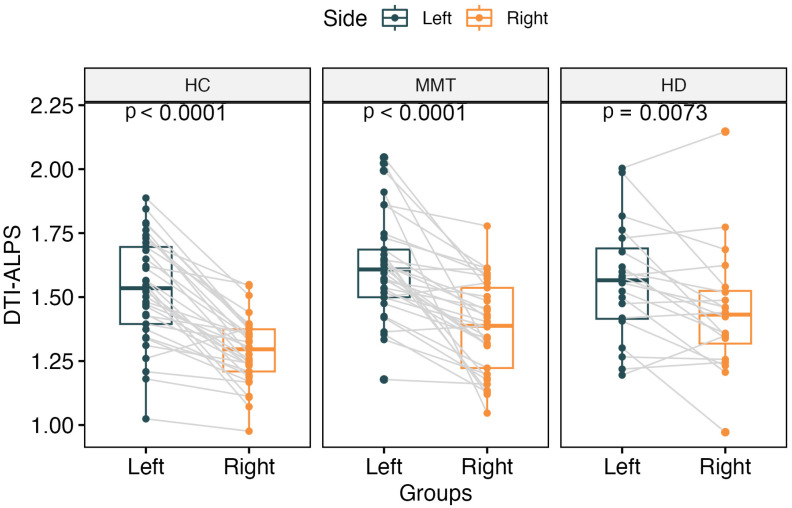
The paired comparisons of bilateral DTI-ALPS for each group. All 3 groups showed significantly higher DTI-ALPS for the left side of the brain than the right side.

The DTI-ALPS index showed a significantly higher value for the left side than the right side of the brain in all three groups’ participants (*t* = 8.84, *p* < 0.0001 for HC group; *t* = 3.00, *p* = 0.007 for HD group; *t* = 6.36, *p* < 0.0001 for MMT group) (Figure 2).

**Figure 3:**
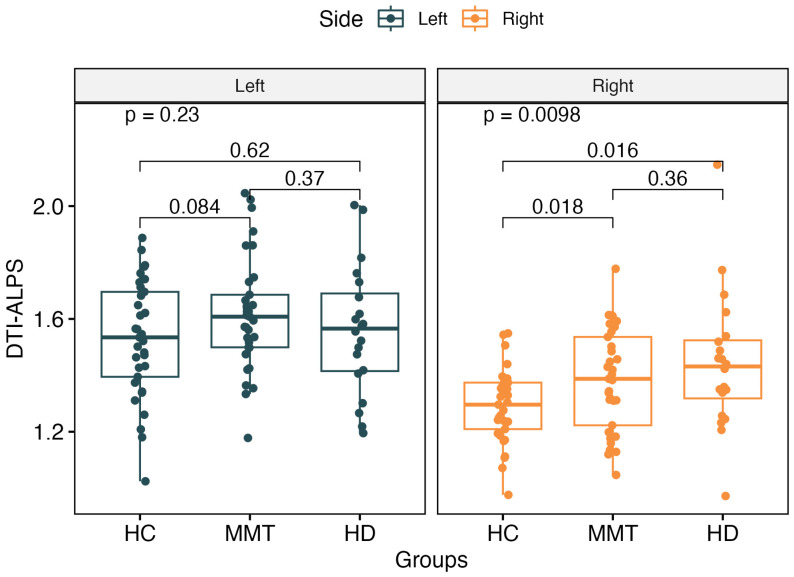
There was no significant difference in the left-side ALPS value among the 3 groups. For the right-side ALPS, the MMT group and HD group showed significantly higher values than the HC group. No significant difference was found between the right-side ALPS of the MMT and HD groups.

The DTI-ALPS index showed no significant difference for the left-side brain among the three groups (*p* > 0.05). For the right-side brain, the DTI-ALPS showed significant differences among the three groups (*F* = 4.89, *p* = 0.0098). The MMT (*t* = 2.44, *p* = 0.018) and HD (*t* = 2.59, *p* = 0.016) groups showed significantly higher values of the right-side ALPS than the HC group, but there was no significant difference between the MMT and HD groups (*F* = 1.48, *p* = 0.23) (Figure 3).

### 3.3. The DivALPS of the Three Groups

**Figure 4:**
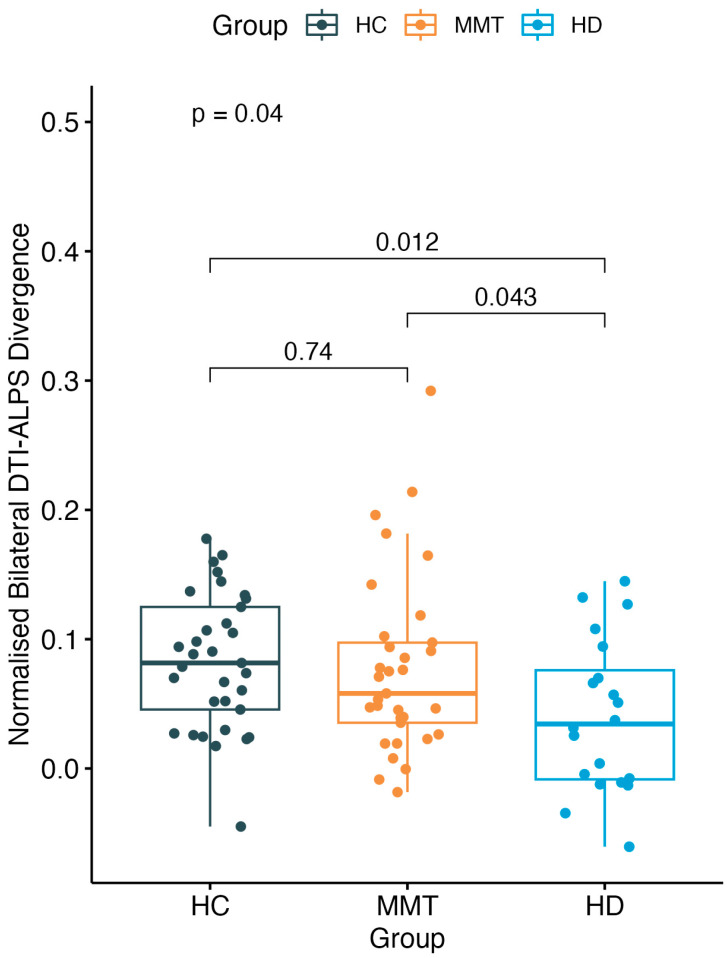
A significant difference was found of normalized bilateral DTI-ALPS divergence (DivALPS) among the 3 groups. Compared with the HC group, the MMT group showed no significant difference in DivALPS. The HD group showed a significantly lower DivALPS than both the HC and MMT groups.

There was a significant difference in DivALPS among the three-group participants (*F* = 3.36, *p* = 0.04). The HD group showed a significantly lower DivALPS value than both the HC group (*t* = −2.65, *p* = 0.012) and MMT group (*t* = −2.09, *p* = 0.043), while the MMT group showed no significant difference compared with the HC group (*t* = −0.34, *p* = 0.74) (Figure 4).

### 3.4. Poisson Regression Result

Poisson regression analysis demonstrated that DivALPS and age had a significant negative relationship with relapse count, and with every 0.1 unit increased in DivALPS, the relapse count decreased by 39.53% (*p* < 0.0001, Exp(B) = 0.6047), and with every 1 year increased in age, the relapse count decreased by 8.58% (*p* < 0.0001, Exp(B) = 0.9142).

## 4. Discussion

In this study, we examined the characteristics of brain glymphatic function in short-term abstinent HD participants and those who underwent stable MMT. We analyzed the relationship between dysfunctional brain GS and relapse risk. Our results demonstrated the negative effects of drug abuse on brain glymphatic function and the protective effects of methadone treatment in facilitating the GS recovery from the state of heroin-induced damage, with divergent effects observed between bilateral brain functions. Regression analysis revealed that bilateral glymphatic function divergence was significantly negatively associated with relapse risk for MMT participants. These findings demonstrate the critical importance of brain GS function in substance addiction.

In this study, the right-side DTI-ALPS index was significantly higher in both the HD and MMT groups compared with the HC group. This suggests the damaging effects of opioid substances on brain GS function. This damage may stem from the disruption of the blood–brain barrier caused by opioid substances. Opioids, such as heroin and methadone, have a direct affinity to pattern-recognition toll-like receptors (TLRs), mainly TLR4 [40], which modulate the opioid neuronal reinforcement-and-reward system [21]. Activation of TLR4 through microglia and astrocyte activation [41] can induce the production and release of pro-inflammatory cytokines, such as interleukin-1β (IL-1β) and tumor necrosis factor-α (TNF-α) [42], contributing to opioid-induced inflammation. TLR4 is constitutively expressed in central-nervous-system vascular endothelial cells, and activation of TLR4 can cause the loss of endothelial-cell junction proteins and dissociation of tight junction complexes, leading to an increase in the permeability of the BBB [43]. The increased permeability of the BBB can stimulate the cerebral lymphatic flow rate to facilitate clearance of the increased molecular load within the brain interstitium [44]. This may explain why heroin promoted the GS function but not in the opposite direction, as reported in a cocaine-induced mice model, where glymphatic pathways were found impaired both structurally and functionally [19]. Enlarged perivascular spaces (EPVS) provide additional evidence of the effects of heroin abuse on the brain. EPVS is a neuroimaging marker of cerebral vessel disease that mainly arises due to inflammation and BBB disruption. Typically, low doses of opium reduce blood pressure through vasodilation and a decrease in sympathetic tone; however, with long-term use, blood-pressure reduction diminishes, and a trend of increased blood pressure supervenes [45]. In a modest-sized postmortem study of perivascular changes, the neurovascular effects of heroin abuse were examined, revealing a higher incidence of inflammatory cells, edema, and hemorrhaging in the perivascular area [46]. Studies have also reported an increased burden of EPVS in both humans and animals who use drugs [47,48], supporting the hypothesis that long-term heroin abuse disrupts BBB permeability, causes inflammation, and increases EPVS, all leading to elevated brain glymphatic-system function. The glymphatic function comprises three different parts: the cerebrospinal-fluid (CSF) influx along the periarterial space, CSF–ISF exchange, and ISF efflux along the perivenous space [49]. The DTI-ALPS index mainly reflects the ISF efflux function along the perivenous space. Based on this inference, our results indicate that heroin abuse may elevate the ISF efflux part of brain GS function. 

Both heroin and methadone act on the same opioid receptors in the brain, primarily the mu-opioid receptors. They produce analgesic and euphoric effects, but they differ in their pharmacokinetics and pharmacodynamics [50]. Heroin is rapidly metabolized into morphine, producing intense but short-lived effects, leading to a cycle of frequent use [51]. Methadone, on the other hand, has a slower onset and longer duration of action, helping stabilize individuals [22]. With its long elimination half-life of 15–55 h [2,52], methadone can accumulate in the body through daily dosages [22]. Although methadone itself has harmful effects on the brain, the damage it causes is much smaller than that of heroin abuse, when under administration. Through the process of “substituting small poison for big poison”, methadone reduces the harm caused by heroin addiction and aids in the repair of heroin-induced brain damage. Heroin addiction correlates with widespread changes in brain structure and function, and these changes are partly reversible through abstinence and treatment. Studies have indicated that MMT improves cognitive function and decreases psychological symptoms in heroin addicts [53,54]. Moreover, methadone has been found to have anti-inflammatory effects in multiple sclerosis [55]. A recent study by Louveau et al. demonstrated the existence of functional lymphatic vessels in the brain, which play a vital role in brain waste clearance and immune surveillance [56]. These findings suggest that methadone’s effectiveness may be due, in part, to its ability to modulate the brain’s lymphatic system. Short-term methadone use has a blood-pressure-lowering effect [45], which may be a direct cause of altering the status of the brain’s lymphatic system. In the long term, due to its substitution effect for heroin, inflammatory responses in the brain are alleviated. Additionally, the recovery of brain tissue microstructure and regeneration of lymphatic capillaries may contribute to the normalization of the brain’s lymphatic system.

The findings of our preliminary study indicate a potentially protective effect of the brain glymphatic system in reducing relapse rates among individuals with heroin addiction undergoing MMT. The closer the bilateral ALPS divergence is to that of normal individuals, the lower the likelihood of relapse. In contrast, Chen et al. used a mouse model of noncontingent cocaine exposure to reveal the structural and functional impairments in the GS pathway [19]. Their findings demonstrated that cocaine treatment reduces the polarity of AQP4, leading to greatly impaired brain GS function in mice [19]. Conversely, Yang et al. showed that AQP4 deficiency potentiated analgesia in acute morphine exposure and attenuated tolerance in mice with chronic morphine exposure; this deficiency is also associated with decreases in opiate-induced drug-seeking and -taking behaviors [57]. These animal model findings differ from our results and may indicate that more complex mechanisms are involved in regulating the human brain GS, including drug-induced inflammation [58], edema [59], and arteriosclerosis [60]. Further research is warranted to clarify these mechanisms and improve our understanding of their interactions. Nevertheless, our study highlights the significance of the brain GS in opioid addiction. One of the key challenges in managing MMT is determining the appropriate medication dosage for each individual. Tailoring MMT dosages to an individual’s needs is critical to minimize relapse risks and promote long-term recovery [61]. Our findings suggest that measuring the brain glymphatic system may provide valuable insights into an individual’s relapse risk. Therefore, by assessing the glymphatic-system functionality in MMT patients, clinicians may be able to develop personalized dosing strategies and improve treatment outcomes. However, because of limited human research surrounding the involvement of the brain GS in regulating relapse behavior, cautious interpretation of our results is necessary.

It should be noted that although different influences on the GS were observed, there is no evidence that heroin and methadone have different mechanisms in their receptor effects. They are both opioid receptor agonists [61]. The difference between heroin and methadone is primarily in lipophilicity, potency, and addictive potential [22,51]. Whether there is direct therapeutic effect of methadone on the GS still needs further investment using some GS modulators or other new methods. Again, these observed differences may come from different chemical structure, pleiotropic effect, influence on the cardiovascular system [62], metabolic changes, and nutritional deficiencies as well. 

In our present study, all three groups exhibited significant hemispheric differences in GS function, with the largest difference in the HC group. This could be explained by the structural differences between bilateral human brains. The first reason may come from the handedness of our subjects, who were all right-handed. The dominant brain side, often coinciding with handedness, tends to have a diameter of superior longitudinal fasciculus bundle, which would influence the measurement of ALPS [63]. Another potential explanation proposed by Zhang et. al was the separate blood supply system of the human brain, as this could be the structural basis of the GS independently functioning bilaterally [64]. Brain asymmetry is commonly found in structure and connectivity in healthy brains and is considered to have associations with neurodegenerative diseases and the relevance to disease severity, progression, and patterns of neuropathology [65]. Our findings suggest DivALPS, which represents the asymmetry of GS function, could be a biomarker of drug abuse disruptions and may have prediction value to the relapse risk of heroin abusers under MMT. However, human GS function can be influenced by many factors, not only the drugs but also the sleep quality [24], blood pressure, eating habits, and even head position [66]. Future studies should validate this GS asymmetry by more-specific non-invasive methods with stringent study control.

There are limitations in the present study. Firstly, the sample size of the HD group was relatively small due to recruitment difficulties in the local district. This situation requires us to interpret the differences between the HD group and other groups with great caution, and future enlarged-sample-size research is needed to validate these results. Secondly, although the DTI-ALPS index is considered to be a representation of the GS functioning, this index can only reveal part of the brain GS, and its measurement methodology restricts its application in only a few axial slices of the brain. To obtain a more comprehensive view of the brain GS, future research could use more-specific methods, such as glymphatic MRI, to compare signal intensity on MRI before and after intrathecal injection of gadolinium. Thirdly, we only recruited male subjects in the present study due to the insufficient number of female addicts. Previous research has indicated that gender influences the brain GS [67]. Therefore, it is necessary to use a more appropriate recruitment strategy and gender-matched groups to verify the reliability and repeatability of our findings in the future.

## 5. Conclusions

In summary, the present study highlights the significance of the GS in opioid addiction and the potential role it plays in reducing relapse rates. While limitations exist, our findings suggest that measuring the brain GS may provide valuable insights into an individual’s relapse risk. Our study offers a potentially straightforward approach to assess individualized relapse risk by measuring the functionality of the GS in MMT patients. This approach has the potential to facilitate the implementation of targeted treatment strategies in clinical practice, ultimately leading to improved treatment efficacy. Further research is needed to validate our findings and elucidate the underlying mechanisms of MMT involved in regulating the glymphatic system.

## Figures and Tables

**Table 1:** Demographic information and clinical characteristic data of HC, HD, and MMT groups.

Characteristic	HC		HD	MMT		T (N1)	P (N1)	F (N2)	P (N2)	T (N2)	P (N2)
	N1 = 48	N2 = 33	N2 = 20	N1 = 51	N2 = 33	MMT-HC	MMT-HC	3 Groups	3 Groups	MMT-HD	MMT-HD
Age (years)	35.42 ± 7.96	31.39 ± 6.18	31.50 ± 7.84	35.76 ± 8.12	30.91 ± 5.34	0.22	0.83	0.07	0.93	-	-
Education (years)	10.1 ± 1.84	10.36 ± 1.85	10.80 ± 2.76	9.67 ± 1.89	10.12 ± 1.98	−1.17	0.27	0.63	0.54	-	-
Nicotine (no. cigarette/day)	16.77 ± 9.67	16.21 ± 9.93	17.25 ± 4.99	19.10 ± 8.71	19.00 ± 9.27	1.26	0.21	0.85	0.43	-	-
Duration of nicotine use (years)	16.75 ± 7.03	13.64 ± 5.97	14.03 ± 5.89	18.24 ± 8.53	14.12 ± 5.77	0.95	0.35	0.93	0.41	-	-
Dosage of heroin use (g/day)	-	-	0.58 ± 0.30	0.41 ± 0.36	0.45 ± 0.40	-	-	-	-	−1.31	0.20
Duration of heroin use (months)	-	-	41.25 ± 25.68	85.57 ± 78.07	58.80 ± 39.30	-	-	-	-	1.69	0.15
Dosage of methadone use (mg/d)	-	-	-	42.51 ± 15.56	42.24 ± 15.59	-	-	-	-	-	-
Duration of MMT (months)	-	-	-	21.03 ± 15.74	20.68 ± 14.80	-	-	-	-	-	-
BDI score	4.46 ± 5.63	4.67 ± 5.34	8.10 ± 9.71	9.53 ± 8.38	10.88 ± 8.86	**3.56**	0.001 *	**5.08**	0.008 *	−0.27	0.79
HAMA score	7.10 ± 8.88	6.46 ± 6.16	10.40 ± 8.18	9.37 ± 8.59	10.70 ± 9.27	1.29	0.2	2.76	0.07	0.55	0.58
Craving score	-	-	1.06 ± 1.51	0.14 ± 0.99	0.19 ± 1.18	-	-	-	-	**−2.10**	0.04 *

* means statistically significant. N1 means the number of participants involved in the comparison between demographic-matched MMT and HC groups. N2 means the number of participants involved in the comparison among three groups: the HC, HD, and MMT groups.

## Data Availability

Data supporting the findings of this study are available from the corresponding author, by contingency of institutional approval, upon reasonable request.
